# Deep-Learning-Aided Detection of Mycobacteria in Pathology Specimens Increases the Sensitivity in Early Diagnosis of Pulmonary Tuberculosis Compared with Bacteriology Tests

**DOI:** 10.3390/diagnostics12030709

**Published:** 2022-03-14

**Authors:** Yoshiaki Zaizen, Yuki Kanahori, Sousuke Ishijima, Yuka Kitamura, Han-Seung Yoon, Mutsumi Ozasa, Hiroshi Mukae, Andrey Bychkov, Tomoaki Hoshino, Junya Fukuoka

**Affiliations:** 1 Department of Pathology, Nagasaki University Graduate School of Biomedical Sciences, 1-7-1 Sakamoto, Nagasaki 852-8501, Japan; zaizen_yoshiaki@med.kurume-u.ac.jp (Y.Z.); yu.kana7173@gmail.com (Y.K.); ishijimas@nagasaki-u.ac.jp (S.I.); ykita@apricot.ocn.ne.jp (Y.K.); hanseungy@gmail.com (H.-S.Y.); 0717mutumi@gmail.com (M.O.); 2 Division of Respirology, Neurology and Rheumatology, Department of Medicine, Kurume University School of Medicine, 67 Asahi-machi, Kurume, Fukuoka 830-0011, Japan; hoshino@med.kurume-u.ac.jp; 3 N Lab Co. Ltd., 1-43-403 Dejima, Nagasaki 850-0862, Japan; 4 Department of Respiratory Medicine, Nagasaki University Graduate School of Biomedical Sciences, 1-7-1 Sakamoto, Nagasaki 852-8501, Japan; hmukae@nagasaki-u.ac.jp; 5 Department of Pathology, Kameda Medical Center, 929 Higashi-cho, Kamogawa, Chiba 296-8602, Japan; bychkov.andrey@kameda.jp

**Keywords:** tuberculosis, artificial intelligence, bronchoscopy, bronchial lavage, mycobacteria

## Abstract

The histopathological diagnosis of mycobacterial infection may be improved by a comprehensive analysis using artificial intelligence. Two autopsy cases of pulmonary tuberculosis, and forty biopsy cases of undetected acid-fast bacilli (AFB) were used to train AI (convolutional neural network), and construct an AI to support AFB detection. Forty-two patients underwent bronchoscopy, and were evaluated using AI-supported pathology to detect AFB. The AI-supported pathology diagnosis was compared with bacteriology diagnosis from bronchial lavage fluid and the final definitive diagnosis of mycobacteriosis. Among the 16 patients with mycobacteriosis, bacteriology was positive in 9 patients (56%). Two patients (13%) were positive for AFB without AI assistance, whereas AI-supported pathology identified eleven positive patients (69%). When limited to tuberculosis, AI-supported pathology had significantly higher sensitivity compared with bacteriology (86% vs. 29%, *p* = 0.046). Seven patients diagnosed with mycobacteriosis had no consolidation or cavitary shadows in computed tomography; the sensitivity of bacteriology and AI-supported pathology was 29% and 86%, respectively (*p* = 0.046). The specificity of AI-supported pathology was 100% in this study. AI-supported pathology may be more sensitive than bacteriological tests for detecting AFB in samples collected via bronchoscopy.

## 1. Introduction

Tuberculosis (TB) is an airborne disease caused by Mycobacterium tuberculosis, resulting in 10 million cases and 1.4 million deaths worldwide annually [1,2]. Additionally, an increased number of nontuberculous mycobacterial (NTM) infections caused by acid-fast bacilli (AFB) have been recently observed in many countries, particularly in Asia [3,4,5,6]. NTM infections affect the lungs, skin, and lymph nodes, which are difficult to manage, cementing it as a significant health problem, similar to TB [7].

AFB infections, such as TB and NTM, are diagnosed either by detection of the mycobacteria through microscopy or culture tests. The most common route of transmission of TB and NTM infections is the respiratory tract; therefore, sputum examination is initially performed [2,3]. Fluorescence microscopy is the most accurate method for sputum smear examination, with a sensitivity of >70% and >50% for TB and NTM, respectively [2,8]. However, in >20–40% of cases, the sputum smear test shows false negatives. In these cases, a lung biopsy using bronchoscopy may be performed, but is not recommended routinely due to its invasive nature [2,3]. However, the sensitivity of transbronchial lung biopsy (TBLB) for the diagnosis of TB only ranges from 42–63%, even when only used to visualize granulomas, not the mycobacteria [9,10].

The effectiveness of artificial intelligence (AI) in pathological diagnosis, especially in oncology, has recently been established [11,12,13,14], and is associated with technological innovations, whole slide imaging (WSI) advancements, and digital capturing of histopathological slides [15,16,17]. AI is desirable in diagnosing mycobacteria histologically, as its comprehensiveness may reduce false negatives; however, obtaining a clear image using WSI is limited by the small size of AFB (0.2–0.6 μm × 1–10 μm). To address this limitation, recent studies have applied AI-based detection of AFB on WSIs [18,19,20]. However, the clinical use of AI in the pathology of mycobacteriosis has not been reported.

This study compared bacteriology and AI-supported pathology in TBLB to validate the clinical usefulness of AI in supporting the pathological diagnosis of mycobacteriosis.

## 2. Materials and Methods

### 2.1. Study Subjects

We selected two representative autopsy cases of pulmonary TB as training data to develop AI-assisted detection of AFB from tissues. In these autopsy cases, we used one typical section where numerous AFBs were detected by Ziehl–Neelsen staining. Additionally, we randomly selected 40 cases who underwent biopsy and had no AFB on Ziehl–Neelsen staining to train other histopathological specimens: eight surgical lung biopsies, 20 TBLBs, six transbronchial mediastinal lymph node needle biopsies, and six bone marrow clots or bone marrow biopsies. Subsequently, 14 consecutive cases that were not used as training data were selected as validation data; these patients underwent bronchoscopy to diagnose mycobacteria. All TBLBs were performed with 2.0-mm forceps, and the number of biopsies was 3–5. We scanned the Ziehl–Neelsen stained tissues at 400× magnification using Motic EasyScan (Motic, Hong Kong, China), and processed them into WSI.

Additionally, patient information, interferon-gamma releasing assay (IGRA) results, bacteriological test results, clinical course, and mycobacteriosis onset were collected from the medical records of the 14 patients used for AI validation.

### 2.2. Annotation

**Figure 1:**
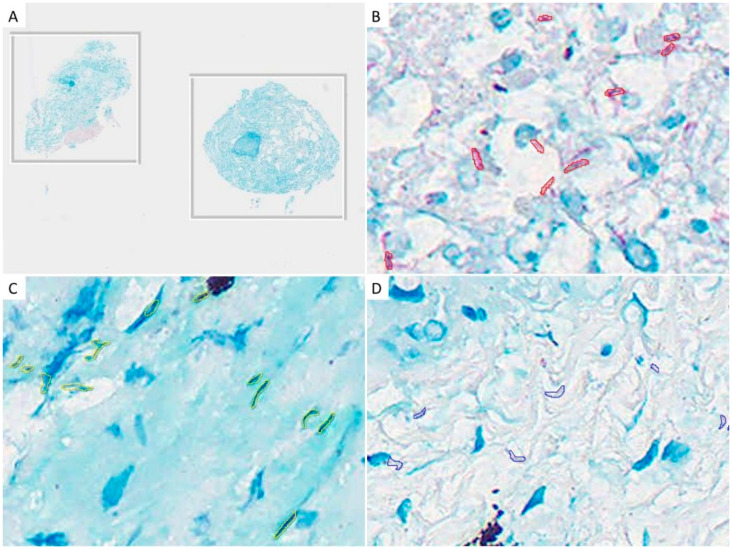
Examples of annotations: (**A**) Ziehl–Neelsen staining tissues, which do not contain acid-fast bacilli (AFB), for the purpose of training the background other than AFB. No annotation was performed; (**B**) We annotated short-rod-shaped bacilli that were stained red in Ziehl–Neelsen staining as AFB; (**C**) Nuclei of type I epithelial cells showing AFB-like morphology, annotated as artifact 1; (**D**) Part of the fibrin-stained purple, annotated as artifact 2.

Annotation was performed by a consensus of three evaluators with expertise in pulmonary pathology (YZ, YK, and JF). We conducted pre-training of the AI using the Ziehl–Neelsen stained tissue. At this stage, we examined artifacts that the AI identified as AFB, but the evaluators judged as false positives compared with WSIs in 40×. Subsequently, we determined two representative patterns of artifacts that the AI misidentified as AFB: artifact 1, blue-to-black-stained nuclei of type I epithelial cells resembling AFB in shape; artifact 2, part of the fibrin and hyaline membrane, which stained pale-purple-to-pale-blue on Ziehl–Neelsen, and shaped similar to AFB. Subsequently, we annotated the AFB and two patterns of artifacts in the WSI as training data for AI (Figure 1). Annotation was performed by importing the WSI tissues into the HALO software (version 3.0; Indica Lab, Corrales, CA, USA), a quantitative image analysis platform. Short bacilli, with a length of 2–10 µm and a width of 0.3–0.6 µm, which stained red on Ziehl–Neelsen staining, were annotated as AFB. In the two autopsy cases used as training data, we annotated 506 AFBs contained in an area of approximately 15 mm^2^. In the 40 biopsy cases, we annotated two patterns of artifacts for all specimens.

### 2.3. Construction of AI-Assisted Pathology

We used HALO AI (CNN, Dense network) to construct an AI-assisted pathology using WSI annotated on the HALO software (version 3.0; Indica Lab, Corrales, CA, USA). To account for the small size of AFB, the WSI resolution was set at 0.25 µm/px. In this study, we tried to build an AI algorithm that could detect AFB by transfer learning from an AI trained to detect malignant diseases, which was constructed in a previous study [21]. The pre-trained HALO AI was integrated with the annotated AFB and two patterns of artifacts. First, 50,000 integrations were performed for AFB and background lung annotations; subsequently, 73,800 integrations were performed, adding the two types of artifacts to build the AI algorithm for AFB detection. The analyzed images were automatically annotated with AFB classified by the AI. The evaluators judged each of these annotations as true or false positives.

This HALO AI used the MXNet engine, DenseNet-121, pre-trained on ImageNet backbone with the firt max pooling layer removed, and with a custom semantic segmentation head. The semantic segmentation head extracted features after each max pooling layer, and before the final fully connected layer. Each feature was passed through (BN-Relu-Conv) block resulting in a 256-channel feature map, which is upsampled by a factor of 2×, and summed with the next higher resolution embedded feature map. During training, auxiliary losses are added at the features extracted at lower resolutions by using a linear layer to predict the correct output, whereas during analysis, only the highest resolution output is used. The training was conducted on 256 × 256 patches at the defined resolution, which were generated by selecting a random class (with equal probability for each class), a random image containing annotations for the selected class (with equal probability), and a random point inside a region of the selected class and image. The patches were cropped surrounding the selected point, and were further augmented with random rotations and shifts in hue, saturation, contrast, and brightness. The model was pre-trained on ImageNet, and thereafter, trained for the defined number of iterations using Adam using an annealed cyclic learning rate (max learning rate, 2 × 10^−4^; minimum learning rate, 1 × 10^−6^; 100 iterations of warmup; initial cycle length, 1000 iterations; cycle length decay, 2.0; cycle magnitude decay, 0.5) (delta of 0.9) with a learning rate of 1 × 10^−3^, which was reduced by 10% every 10,000 iterations along with an L2 regularization of 1 × 10^−4^. During analysis, the tile size was increased to 896 × 896 without significantly altering the output, while increasing the performance.

### 2.4. Validation of the AI-Assisted Pathology

**Figure 2:**
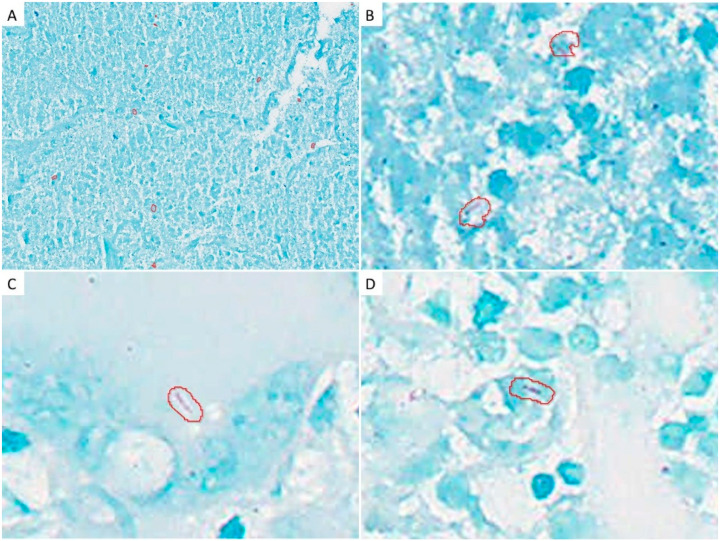
Examples of acid-fast bacilli (AFB) recognized by AI in the study cohort: (**A**) AFB observed in a relatively large group (middle magnification); (**B**) High magnification; (**C**) AFB was observed sporadically at high magnification; (**D**) An example in which AI determined AFB, but the pathologist determined it as a false positive.

In the 42 patients who underwent bronchoscopy due to suspected mycobacteriosis, or to rule out mycobacteriosis, one pathologist (JF) first evaluated the Ziehl–Neelsen stained tissue using manual light microscopy to diagnose the presence of AFB. Subsequently, the WSIs of these patients were comprehensively analyzed using the constructed AI to identify AFB. For each annotation the AI recognized as AFB, six evaluators (YZ, YK, SI, YK, HSY, and JF) determined by consensus whether it was a true or false positive. Similar to ordinary pathological diagnosis, a positive slide contained at least one true positive annotation, and a negative slide contained no true positives (Figure 2). The AI-supported pathology results were compared with bacteriological tests, such as mycobacterium smear, culture, and nucleic acid amplification test (NAAT) using bronchial lavage fluid (BLF). TBLB for pathological evaluation, and BLF collection for bacteriology, were performed at the same bronchus branch. Mycobacterium smear, culture, and NAAT were performed using auramine-rhodamine staining, mycobacteria growth indicator tubes (Nippon Becton Dickinson Co., Ltd. Tokyo, Japan), and polymerase chain reaction (Loopamp; Eiken Chemical Co., Ltd. Tokyo, Japan), respectively. Additionally, we collected information regarding mycobacteriosis development from the patients’ medical records. Chest computed tomography (CT) was performed, and two experienced pulmonologists (YZ and MO) determined the following imaging features of mycobacteriosis [22]: nodular shadows, bronchiectasis, consolidation, cavity formation, and lymph node enlargement.

The histopathological and bacteriological examinations sensitivities were compared with the final diagnosis (ground truth), which defined true positives as patients with a confirmed diagnosis of mycobacteriosis using bronchoscopy, or patients who developed mycobacteriosis during the follow-up period after bronchoscopy; all other patients were considered true negatives. Mycobacteriosis was diagnosed by bacteriological tests according to the guidelines [2,3].

### 2.5. Statistical Analysis

The patient characteristics data are presented as median values with a 25–75% interquartile range. The difference between the bacteriological and pathological test sensitivity was analyzed using the McNemar test. Statistical significance was defined as *p* < 0.05, and all statistical analyses were performed using JMP 16.0 (SAS Institute, Cary, NC, USA).

All numerical data are presented as median values with a 25–75% interquartile range. Statistical significance of the difference between two or three groups was analyzed using the Wilcoxon rank-sum test, Mann–Whitney U test, or Fisher’s exact test, where applicable. Statistical significance was defined as *p* < 0.05, and all statistical analyses were performed using JMP 14.0 (SAS Institute, Cary, NC, USA).

## 3. Results

### 3.1. Patient Characteristics

The information of the 42 patients used for AI validation study is shown in Table 1. All patients underwent bronchoscopy as the clinical course, and other tests did not provide a definitive diagnosis. In the mycobacteriosis group, seven patients (44%) each were diagnosed with TB and NTM infection; two patients (12%) were not confirmed by subsequent follow-up, but mycobacteriosis was strongly suspected. Chest CT scan revealed multiple nodular shadows in 13 (81%) patients, consolidation in nine (56%), cavity formation in three (19%), and bronchiectasis in six (38%) in the mycobacteriosis group.

### 3.2. Bacteriological Examination by Bronchoscopy

Using BLF, antibacterial smear, antibacterial culture, and NAAT were performed for all patients (Table 2). In the non-mycobacteriosis group, no cases were positive in the bacteriological tests. In the mycobacteriosis group, bacteriological examination of BLF showed a positive smear test in four patients (25%); among them, Mycobacterium tuberculosis and NTM were detected in one and three patients, respectively. Mycobacterium culture was positive in seven (44%) patients, among which, two had TB, and five had NTM infection. Mycobacterium NAAT test was positive in two NTM infections, in addition to the positive cases in culture. Among the 16 mycobacteriosis patients, 9 (56%) were positive for bacteriological tests. Seven cases (44%) had positive smear or culture tests which led to a definitive diagnosis.

### 3.3. Pathological Examination

Initially, one pathologist screened all patients to detect the presence of AFB using oil immersion microscopy, without AI; among them, two were positive for AFB. Subsequently, all patients underwent AFB evaluation using WSI with AI support, which identified 11 patients positive for AFB. Additionally, the two patients the pathologist diagnosed with AFB were also identified by the AI-supported pathology.

### 3.4. Comparison between Pathology, Bacteriology, and Final Diagnosis

The results of the bacteriological tests and AI-supported pathology in the mycobacteriosis and non-mycobacteriosis groups are shown in Table 3. Among all mycobacteriosis, seven cases (44%) showed positive results in smear or culture, which confirmed the diagnosis. Nine cases (56%) were positive in bacteriological tests, including NAAT. AI-supported pathology was positive in 11 cases (69%). There was no significant difference between the results of bacteriological tests and AI-supported pathology in all mycobacteriosis cases.

When limited to TB, AI-supported pathology had significantly better sensitivity compared with bacteriological testing using bronchoscopy (86% vs. 29%; *p* = 0.046). In NTM infection cases, AI-supported pathology was less sensitive than in TB cases, as only three cases (43%) were positive (*p* = 0.046).

### 3.5. Comparison of the Number of AFB Detected by AI and Bacteriological Tests

This study also compared the number of AFB observed using bacteriological tests, radiological findings, and the final diagnosis (Table 4). Two patients demonstrated >100 AFB in their histopathological specimens, and had positive bacteriological tests, such as smear test, culture test, and NAAT. Additionally, among the seven patients with <10 AFB, three were diagnosed with TB, two with NTM, and two were under follow-up. In the seven patients with mycobacteriosis and no evidence of progressive disease, such as consolidation or cavitary formation on radiology, the sensitivity of AI-supported pathology was 86%, which was significantly higher than that of bacteriological tests (29%, *p* = 0.046).

## 4. Discussion

This study validated the usefulness of AI-supported pathology in diagnosing mycobacteriosis. AI-supported pathology in TBLB had higher sensitivity compared with bacteriological tests using BLF. This study included 42 patients for validation, and showed AI-supported pathology had significantly higher sensitivity compared with bacteriological tests when limited to TB. Furthermore, the specificity of AI-supported pathology in this study was 100%. This study showed the usefulness of AI in comprehensively screening AFB, which is frequently missed using bacteriology.

This study also shows the clinical utility of AI-supported pathology. Generally, the sensitivity of the pathology for mycobacteriosis is approximately 50–80% lower compared with bacteriological tests [2,23]. However, this study detected AFB using comprehensive AI screening despite negative results when AI was not used. Notably, Pantanowitz et al. reported easier diagnosis using AI-assisted review, due to its higher sensitivity, negative predictive value, and accuracy compared with light microscopy and WSI evaluation without AI [24]. The current study showed that pathological diagnosis may be better at detecting AFB than traditionally indicated.

Furthermore, we found a low positive predictive value (PPV) of bacteriological tests (1/7, 14%) when AFB was low in pathological specimens, even when using AI comprehensive analysis. Additionally, the PPV of bacteriological tests was high (78%) in nine patients diagnosed with mycobacteriosis who demonstrated cavitary lesions or consolidation on radiology, which are suggestive of progressive disease; the PPV was low (29%) in the seven patients with mycobacteriosis with no signs of progressive disease on radiology, which is consistent with a previous study that found a higher AFB detection rate when cavity formation was found in TB [25]. In contrast, AI-supported pathology showed an 86% higher sensitivity than bacteriological tests in early-stage mycobacteriosis with no cavitary lesions or consolidation on CT, suggesting the superiority of AI-supported technology in detecting early-stage bacterial infections. Particularly, multiple modalities, using AI-supported pathology and bacteriological tests, may be useful in diagnosing early-stage mycobacteriosis, especially when using bronchoscopy.

Few reports examine the usefulness of bronchoscopy, especially TBLB, in mycobacteriosis. Bronchoscopy in TB diagnosis is mainly recommended for bronchial brushing and BLF, whereas TBLB is recommended for patients who require rapid diagnosis [2]. However, some studies on HIV patients only had TBLB as the available tool to diagnose TB in 10–23% of patients [9,26]; these studies considered TB diagnosis using characteristic histological findings, such as caseating granuloma, even if no AFB was found. Among those diagnosed with TB, AFB was observed in 14–57% of patients [2].

This study demonstrated that comprehensive AI analysis detected AFB in many patients, even if pathologists without AI were unable to. AI-supported pathology may detect AFB in patients that can only be diagnosed by TBLB. The small size of the TBLB specimen required only <1 min of WSI examination of the specimen. Therefore, AI-supported pathological diagnosis can be performed without changing daily practice and prolonging diagnostic time. Additionally, NAAT and whole-genome sequencing (WGS) have recently been used to determine drug resistance [27,28,29]. One study found the usefulness of reverse transcription-polymerase chain reaction in identifying species using tissue biopsy [30]. The combination of NAAT and WGS in AI-supported pathology-confirmed tissue may also allow for the measurement of drug susceptibility, which is important in developing treatment strategies, even in negative antibiotic culture tests. Additionally, performing NAAT and WGS after identifying tissue confirmed to have AFB by AI-supported pathology may be more cost-effective than performing these tests on all specimens.

This study showed the superior sensitivity of comprehensive AI analysis compared with a pathologist in detecting AFB. To screen all tissue samples with AI, it is necessary to process them into WSI. However, AFB detection by AI is greatly affected by the availability of high-quality images during WSI processing, which may greatly reduce AFB detection sensitivity. The usefulness of AI-supported pathology can be cited through the disadvantages and advantages of an increased AFB detection rate. This study demonstrated the usefulness of AI-supported technology through its advantages. However, other deep learning models and improved scanning quality for WSI may be used to build more useful models.

This study has several limitations. First, we only studied WSI produced by a single scanner and deep learning in one model. It may be important to compare this AI with other algorithms for the detection of AFB in the future. Second, only autopsy cases of TB were used as AFB-positive training data to construct the AI; additionally, NTM patients were not used as training data due to the rarity of biopsies with numerous microorganisms. In this study, the sensitivity of AI-supported pathology in NTM infection was very low. Different results may be obtained if NTM infection is added as training data. Third, the AI detects a high frequency of false-positive AFB: approximately 200–500 false positives for every true positive. We tried to reduce the number of false positives by recognizing the typical artifacts separately; however, numerous false positives remained. Previous reports described methods of combining multiple AI algorithms to improve AI diagnostic accuracy. Xiong et al. reported that compared with a pathologist, the AI diagnostic sensitivity using a CNN model was 86%, but increased to 98% after combining random forest classifiers [18], which was consistent with another study [19]; these reports show the possibility of reducing false-positive AFBs by combining AI algorithms. However, attempting to reduce false positives may cause true positives to become false negatives. The clinical utility of AI-assisted pathology requires a low false-negative rate.

## 5. Conclusions

AI-supported pathology may be more sensitive than bacteriological tests for detecting AFB in samples collected by bronchoscopy, especially in the early stages of the disease. Furthermore, combining multiple modalities may be necessary to detect AFB, since bacteriological tests may be insufficient.

## Figures and Tables

**Table 1:** Patient characteristics.

	Mycobacteriosis	Non-Mycobacteriosis
Number	16	26
Age	71 (58–76)	63 (46–69)
Sex: Male	8 (50%)	11 (42%)
Serological test: positive		
IGRA	8 (50%)	0 (0%)
anti-MAC antibody *	5 (31%)	1 (4%)
HRCT findings		
Nodular shadow	13 (81%)	16 (62%)
Consolidation	9 (56%)	10 (38%)
Cavity formation	3 (19%)	3 (12%)
Bronchiectasis	6 (38%)	6 (23%)
LN enlargement	3 (19%)	10 (38%)
Final Diagnosis		
TB	7 (44%)	0 (0%)
MAC infection	7 (44%)	0 (0%)
Follow-up †	2 (12%)	0 (0%)
Sarcoidosis	0 (0%)	10 (38%)
Other infectious disease	0 (0%)	4 (15%)
Interstitial lung disease	0 (0%)	3 (12%)
Other ‡	0 (0%)	9 (35%)

HRCT, high-resolution computed tomography; IGRA, interferon gamma releasing assay; LN, lymph node; MAC, Mycobacterium avium complex; TB, tuberculosis. * Anti-glycopeptidolipid core IgA antibody. † Mycobacteriosis group included the follow-up cases with a strong suspicion of mycobacteriosis strongly. ‡ These cases were diagnosed with bronchiectasis, cryptogenic organizing pneumonia, or diffuse panbronchiolitis. Some cases withdrew during follow-up.

**Table 2:** Results of the bacteriological and pathological examinations.

	TB	NTM Infection	All Mycobacteriosis	Non-Mycobacteriosis
Number	7	7	16	26
Bacteriological tests				
Smear	1 (14%)	3 (43%)	4 (25%)	0 (0%)
Culture	2 (29%)	5 (71%)	7 (44%)	0 (0%)
NAAT	2 (29%)	7 (100%)	9 (56%)	0 (0%)
Pathological tests				
Pathology w/o AI	2 (29%)	0 (0%)	2 (13%)	0 (0%)
Pathology with AI	6 (86%)	3 (43%)	11 (69%)	0 (0%)

AI, artificial intelligence; NAAT, nucleic acid amplification test; NTM, nontuberculous mycobacteriosis; TB, tuberculosis; w/o, without.

**Table 3:** Comparison of bacteriological tests and AI-supported pathology.

	Smear or Culture	All Bacteriology	Pathology with AI	*p*-Value *	*p*-Value †
TB (*n* = 7)	2 (29%)	2 (29%)	6 (86%)	**0.046**	**0.046**
NTM infection (*n* = 7)	5 (71%)	7 (100%)	3 (43%)	0.317	**0.046**
All mycobacteriosis (*n* = 16)	7 (44%)	9 (56%)	11 (69%)	0.206	0.527
Non-mycobacteriosis (*n* = 26)	0 (0%)	0 (0%)	0 (0%)	N/A	N/A

AI, artificial intelligence; NTM, nontuberculous mycobacteriosis; TB, tuberculosis. * “smear or culture” vs. “Pathology with AI” † “All bacteriology” vs. “Pathology with AI”.

**Table 4:** Results of radiological, bacteriological, and pathological examinations in mycobacteriosis cases.

No	Age	Sex	Dx	Bacteriological Test			Pathological Test			Radiological Findings
				Smear	Culture	NAAT	Path w/o AI	Path with AI	AFB Count *	
1	64	F	TB	+	+	+	+	+	3+	nodular shadow, consolidation, cavity
2	57	M	TB	−	+	+	+	+	2+	consolidation, LN enlargement
3	30	M	TB	−	−	−	−	+	1+	nodular shadow, consolidation
4	80	F	TB	−	−	−	−	+	1+	nodular shadow, LN enlargement
5	78	F	TB	−	−	−	−	+	2+	nodular shadow
6	55	M	TB	−	−	−	−	+	1+	nodular shadow, LN enlargement
7	30	M	TB	−	−	−	−	−	−	nodular shadow, bronchiectasis
8	70	M	NTM	+	+	+	−	+	3+	nodular shadow, bronchiectasis
9	74	F	NTM	+	+	+	−	+	1+	consolidation, cavity, bronchiectasis
10	62	F	NTM	−	−	+	−	+	1+	nodular shadow, bronchiectasis
11	71	F	NTM	+	+	+	−	−	−	nodular shadow, consolidation, bronchiectasis
12	78	F	NTM	−	+	+	−	−	−	nodular shadow, consolidation
13	70	M	NTM	−	+	+	−	−	−	nodular shadow, consolidation
14	76	F	NTM	−	−	+	−	−	−	nodular shadow, consolidation, cavity
15	74	M	f/u	−	−		−	+	1+	nodular shadow
16	71	M	f/u	−	−		−	+	1+	consolidation, bronchiectasis

AI, artificial intelligence; COP, cryptogenic organizing pneumonia; Dx, diagnosis; f/u, follow-up; LN, lymph node; NAAT, nucleic acid amplification test; NTM, nontuberculous mycobacteriosis; Path, pathology; TB, tuberculosis; w/o, without. * (−): AFB negative, (1+): Less than 10 AFBs observed per biopsied tissue, (2+): 10–100 AFBs observed per biopsied tissue, (3+) >100 AFBs observed per biopsied tissue.

## Data Availability

The data that support the findings of this study are available from the corresponding author upon reasonable request.
